# Second primary lung carcinoma in patients with first primary breast carcinoma: two case reports and review of the literature

**DOI:** 10.4076/1757-1626-2-7993

**Published:** 2009-07-24

**Authors:** Shi-Ping Luh, Ching-Chung Chiang, Mao-Te Chuang

**Affiliations:** 1 Department of Surgery, Chia-Yi St Martin De Porres Hospital and Institute of Biomedical Engineering, National Chiao-Tung UniversityNo. 565, Sec 2, Da-Ya Rd, Chia-Yi CityTaiwan; 2 Chia-Yi Christian Hospital and Institute of Biomedical Science, National Chung-Cheng UniversityNo. 539, Chung-Shiao Rd, Chia-Yi CityTaiwan

## Abstract

**Introduction:**

Patients undergoing complete treatments of breast carcinoma can be found with pulmonary nodules during the follow up period. Either metastasis from breast carcinoma or second primary bronchogenic carcinoma should be considered as a possible diagnosis.

**Case presentations:**

Two female patients with ages of 66 and 64, underwent modified radical mastectomy for breast carcinoma 5 and 2 years ago, were found with single pulmonary nodule, 1.0 cm and 0.8 cm from the left lower and right upper lobe. There was no other site of metastasis being noted after systemic survey.

Wedge resections through video assisted thoracic surgery were performed and one of them underwent lobectomy and mediastinal lymph node dissection after the primary lung carcinoma being proved pathologically (thyroid transcription factor 1 and cytokeratin 7 positive). The dissected lymph node in this patient is negative for malignancy. They underwent low dose chemotherapy postoperatively because of increased risk of tumor occurrence for these patients. Patients with smoking or irradiation history usually favor the diagnosis of second primary lung carcinoma. However, these two treated breast carcinoma cases, which didn’t have smoking or irradiation history, developed second primary lung carcinomas. It is relatively rare reported before.

**Conclusions:**

Pulmonary nodules in patients with prior breast carcinomas were usually regarded as metastatic lesions. However, the possibility of second primary still cannot be excluded, especially to the solitary type. Video assisted thoracic surgery can provide early and accurate diagnosis as well as effective treatment.

## Introduction

Pulmonary nodules that appear in a patient with prior malignancy may be a metastasis or a second primary lung cancer [1]. Lung cancer is so prevalent in the world that the possibility of a second primary lesion in the above situation, especially a solitary nodular type, is much higher than before. Many factors, such as the type of prior malignancies, the choice of treatment strategies especially the field and dose of irradiation, the smoking history and the radiographic characteristics will influence the possibility of diagnosis [2]. Herein two treated breast carcinoma cases without prior smoking or irradiation history, developed second primary lung carcinomas, which were relatively rarely presented before.

## Case presentations

### Case report 1

**Figure 1.**
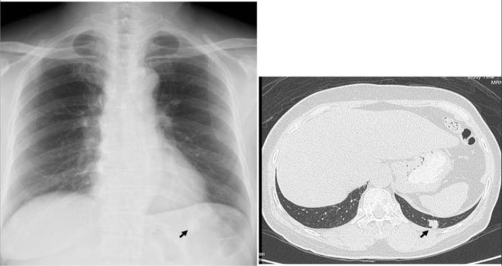
Chest X-ray and CT in Case 1 revealed a 1 cm nodular shadow over the supra-diaphragmatic area of the left lung.

**Figure 2.**
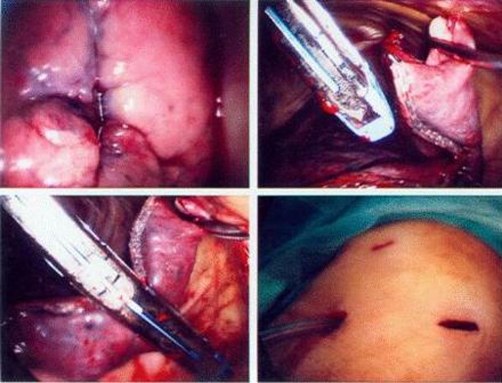
Video-assisted thoracoscopic surgery in Case 1. **(A)** the nodule revealing visceral pleural retraction sign. **(B)** and **(C)** This nodule was resected by Endo-GIA stapling and division. **(D)** The thoracoscopic wound (three ports design).

A 66-year-old non-smoking Taiwanese woman had received modified radical mastectomy (MRM) for her left breast carcinoma 5 years ago. No axillary lymph nodal metastasis (0/11) was noted. The estrogen and progesterone receptors are negative, and Her2/neu shows over-expression (2+) on the resected specimen. She underwent postoperative adjuvant chemotherapy with 6 courses of intravenous epirubicin and fluorouracil, oral UFUR control for 24 months and Nolvadex for 5 years. No local irradiation was administered before. The clinical course was smooth postoperatively, until a pulmonary nodule was accidentally found on the Chest X-ray several days ago. Chest computed tomography (CT) revealed a 1 cm mild enhancing nodule in posterior segment of left lower lobe of lung with spiculated margin (Figure 1). The serum carcino-embryonic antigen (CEA) level was within normal range (0.5 ng/ml). Pulmonary primary carcinoma or metastasis is highly suspected and thus she underwent tumor wedge resection through video-assisted thoracoscopic surgery (VATS) (Figure 2). The pathological report revealed a TTF1 and CK7 positive primary lung adenocarcinoma. Thus she underwent left lower lobectomy and mediastinal lymph nodes dissection. No residual tumor or lymph nodes metastasis was noted on histopathological examination. She underwent oral UFUR control postoperatively and her condition is stable without evidence of tumor recurrence or metastasis.

### Case report 2

**Figure 3.**
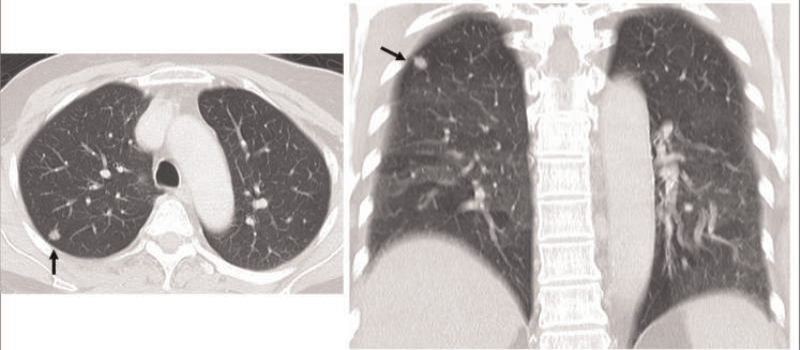
Chest X-ray and CT in Case 2 revealed a 0.8 cm nodular shadow over the B2 segment of the right upper pulmonary lobe.

**Figure 4.**
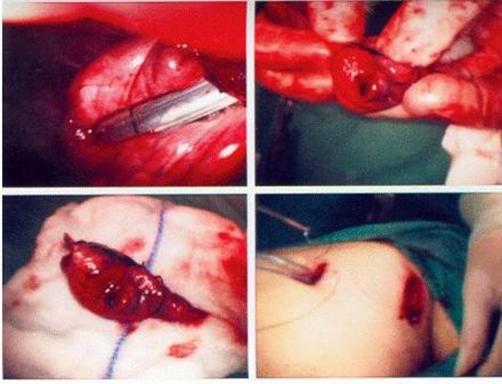
Video-assisted thoracoscopic surgery in Case 2. **(A)** the nodule was localized by hand palpation and then resected by endo-GIA. **(B)** and **(C)** nodule can be seen on the cut surface of the resected specimen. **(D)** The thoracoscopic wound (one scope port and a 3 cm utility incision).

A 64 year old non-smoking Taiwanese woman received MRM for her right breast carcinoma 2 years ago. No axillary lymph nodal metastasis (0/21) was noted. The estrogen and progesterone receptors are negative, and Her2/neu shows over- expression (3+) on the resected specimen. She underwent oral chemotherapy with UFUR and hormone therapy with Nolvadex postoperatively. However, a tiny pulmonary nodule was noted accidentally on the chest X-ray after two years of follow up. Chest CT revealed a 0.8cm nodule with smooth margin over the right upper lobe of lung (Figure 3). She underwent VATS wide excision under the impression of pulmonary metastasis from breast carcinoma (Figure 4). However, the pathological report revealed a TTF1 (+) and CK7 (+) bronchoalveolar carcinoma with mucinous variant and the safety margins were over 2 cm. After discussion with her families, she was undergoing postoperative intravenous NC (navelbine/cisplatin) regimen for four courses. She was stable on the 4 months of follow up period.

## Discussion

Breast irradiation will increase the risk of second primary lung carcinoma, and a multiplicative effect was observed in smokers. For both smokers and nonsmokers, this effect only existed in the ipsilateral lung [3,4]. Modern techniques, however, significantly decrease the radiation dose to the lungs, which may decrease the risk of lung cancer [5]. Galper S et al (2002) reviewed about 1800 stage I or II breast carcinoma patients who underwent surgery followed by adjuvant radiotherapy [6]. Second non-breast malignancies occur in a substantial minority (8%) of patients treated with conservative surgery and radiotherapy, and the absolute excess risk compared with the general population is very small (1%) and this excess risk is only evident after 5 years. Obedian E et al (2000) also found that lumpectomy followed by modern irradiation technique will not increase the risk of second malignancies compared with mastectomy [7]. Fowble B et al (2001) found that in breast carcinoma patients undergoing lumpectomy and irradiation treatment, contralateral breast cancer will occur more likely in young patients, and the non-breast cancer second malignancy will more commonly occur in older ones [8]. The correlation between cigarette smoking and bronchogenic carcinoma still exists, but never so strong as before. In recent years, more and more non-smokers suffered from lung cancers, especially the adenocarcinoma. However, these above rules are contradictory to our two patients with prior breast carcinoma and second primary lung carcinoma. Both of them were old aged non-smokers, and never received irradiation before.

Prior tumor type and number of nodules will influence the possibility of diagnosis in patients with pulmonary nodules at the follow up period. Solitary pulmonary nodule (SPN) is more likely to be a second primary lung carcinoma than multiple nodules. SPN in patients with carcinomas of the head and neck, bladder, breast, cervix, bile ducts, esophagus, ovary, prostate, or stomach, were more likely to be a primary bronchogenic carcinoma than a metastatic lesion. SPN in patients with carcinomas of the colon, salivary glands, adrenal gland, thyroid, thymus, kidney or uterus had fairly even odds in the above two possibilities. However, SPN will be more likely a metastatic lesion than a primary tumor in patients with melanoma, sarcoma, or testicular carcinoma [1,9,10].

Solitary pulmonary nodule (SPN) occurs in about 1 per 500 chest radiographs. The most important goal of its diagnosis is to differentiate benign from malignant lesions. Computed tomography (CT) should be considered for all patients with SPNs, because it can provide more information for subsequent management strategies [11]. Nodule morphology might provide valuable information to judge the type of lesion. Smooth margins would be more indicative of a benign lesion or metastasis, whereas irregular margins might be suggestive of a new lung cancer [12]. The existence of regional lymph nodes, the number of nodules and their relationships with adjacent structure can also give information to discern these two possibilities. Further imaging evaluation, such as positron-emitted tomography (PET), is generally not recommended because of its limited specificity for the diagnosis of SPN [13].

Tissue diagnosis is usually required except that the possibility of malignancy is very low. Needle biopsy through the guidance of CT or sonography can be considered but with lower specificity and result in significant complications, such as pneumothorax and hemothorax [14]. Total excision of the SPN through video-assisted thoracic surgery or thoracotomy is usually indicated for specific diagnosis and definite therapy [15,16]. Many localization techniques, such as hooks, coils, and radiotracer markers can be used to facilitate the subsequent resection procedures [17]. However, in our experience most of the peripheral nodule, even though sub-centimeter in size, can be located by imaging and confirmed by finger palpation.

The distinction of a primary lung carcinoma from a metastatic lesion is important, because the treatment and prognosis differ for patients with these malignancies. In histological sections, the existence of acini, lepidic growth, nuclear pseudoinclusions, and scar favor the diagnosis of primary lung adenocarcinoma; on the contrary, comedonecrosis, solid nests, trabecular architecture, and cribriform growth can be identified in metastatic breast carcinoma [18]. Such a distinction can also be achieved by detection of special markers on the histological specimens, such as Thyroid transcription factor-1 (TTF-1) or mammaglobin 1 [19,20]. TTF-1, which was used in our presented cases, is a protein that regulates transcription of genes, and thus it is used as a marker to determine if a tumor arises from the lung or thyroid [19]. TTF1 is usually positive in pulmonary adenocarcinoma or small cell carcinoma. However, rarely does it be positive in the squamous or large cell carcinoma of lung. TTF1 will also express in tumors other than lung and thyroid, such as ovarian or endometrial carcinoma [21,22]. PE-10, a monoclonal antibody against components of human surfactant proteins, and cytokeratin (CK) 7 and 20, are also fairly specific markers for the diagnosis of primary lung tumors [23]. The survival of patients with second primary lung cancer depends on the timing of diagnosis and the staging during resection [24], and thus early detection of pulmonary nodule by image follow up and surgical intervention at early stage are very important for these patients.

## Conclusions

Pulmonary nodules in patients with prior breast carcinomas were usually regarded as metastatic lesions. However, the possibility of second primary lesions still cannot be excluded, especially to the solitary type. VATS can provide early and accurate diagnosis as well as effective treatment.

## Abbreviations

CK: cytokeratin
CT: computed tomography
PET: positron emitted tomography
SPN: solitary pulmonary nodule
VATS: video-assisted thoracoscopic surgery
